# Case Series: Reactivation of Herpetic Keratitis After COVID-19 mRNA Vaccination During Herpetic Prophylaxis

**DOI:** 10.3390/vision9030063

**Published:** 2025-07-28

**Authors:** Michael Tsatsos, Efthymia Prousali, Athanasios Karamitsos, Nikolaos Ziakas

**Affiliations:** 2nd Ophthalmology Department, Aristotle University of Thessaloniki, Papageorgiou Hospital, Ag. Pavlou 76, 56429 Thessaloniki, Greecengziakas@auth.gr (N.Z.)

**Keywords:** herpetic keratitis recurrence, herpetic prophylaxis, COVID-19, vaccination

## Abstract

This report presents two cases of herpes simplex keratitis recurrence after COVID-19 mRNA vaccination in patients on herpetic prophylaxis due to recurrent herpetic keratitis. A 58-year-old man with a history of a previous penetrating keratoplasty presented with blurred vision and evidence of corneal endothelitis 48 h after the first dose of the m-RNA vaccination, and a 24-year-old male student came with a dendritic ulcer 72 h post first vaccination dose. The original prophylactic treatment of 400 mg of acyclovir twice daily was increased to five times per day for a week for both patients. The grafted patient additionally received an increase in Dexamethasone 0.1% from twice daily to four times a day. Improvement was noted within two days and documented at the weekly review, during which both patients returned to their prophylactic antiviral regime without further recurrence. At the time of their second dose of vaccination, both patients followed the same regime with an increase in treatment as per the first dose of vaccination without recurrence. Our findings suggest that patients with recurrent herpetic disease receiving prophylactic treatment need close monitoring when experiencing even subtle symptoms of recurrence and may benefit from an increase in their dose to therapeutic levels during the first days after the COVID-19 mRNA vaccination.

## 1. Introduction

Herpes simplex virus (HSV), an enveloped DNA virus, is a ubiquitous pathogen that commonly infects humans. After primary infection, Herpes simplex virus remains in a latent form in the nervous tissue (sensory and autonomic neurons of the trigeminal and dorsal ganglia) [1]. Studies have also confirmed the presence of HSV in its latent form in the cornea; however, this remains controversial [2]. Herpes simplex infection can involve both the anterior and posterior segments of the eye, but most commonly it is seen as keratitis, which is responsible for a range of ocular manifestations, from superficial epithelial disease to stromal keratitis and endotheliitis [3]. The virus can be reactivated periodically in response to various stimuli, including immunosuppression, psychological stress, fever, ultraviolet exposure, sunlight, fatigue, lack of sleep, and hormonal imbalance [3,4].

With the global COVID-19 vaccination program being at least partly reinstated, it is important to highlight two cases of herpetic keratitis reactivation following COVID-19 mRNA vaccination, despite the ongoing herpetic prophylaxis.

## 2. Case Reports

We report two cases of herpes simplex virus keratitis reactivation following COVID-19 mRNA (Moderna Inc., Cambridge, MA, USA and Pfizer Inc., Kalamazoo, MI, USA) vaccination, despite undergoing herpetic prophylactic treatment. Both patients were on oral antiviral treatment with aciclovir 400 mg twice a day. The grafted patient was also on topical G. Dexamethasone 0.1% (Thea, Clermont Ferrand, France) twice a day. Both experienced flu-like symptoms, including fatigue, muscle aches and mild fever, after their first COVID-19 mRNA vaccine. Informed consent was obtained for both patients. Ethical approval was waived because this was a retrospective clinical observation that did not alter the treatment protocol.

### 2.1. Case 1

A 58-year-old man had a Penetrating Keratoplasty (PK) in his left eye for corneal stromal scarring due to recurrent herpetic stromal keratitis and corneal endotheliitis for the past 30 years. The patient was in good general health and utilized oral hydroxyurea 500 mg once a day and acetylosalicylic acid 75 mg once a day for essential thrombocythemia.

The patient underwent Penetrating Keratoplasty three months before first vaccination and was still receiving oral herpetic prophylaxis initiated post-surgery, along with topical 0.1% dexamethasone (Thea, Clermont Ferrand, France) used twice daily.

Our corneal graft post-operative follow-up protocol includes assessments on the first day, the first week, and subsequently on a monthly basis for the initial six months. After this period, follow-ups transition to every three months for the remainder of the year. The patient was evaluated for his three-month follow-up (which was only a week before his vaccination), presenting with a visual acuity of 0.0 LogMAR (1.0 Snellen) ETDRS in the right eye and 0.3 LogMAR (0.5 Snellen) in the left eye. The ocular examination was normal, showing no signs of intraocular inflammation.

**Figure 1:**
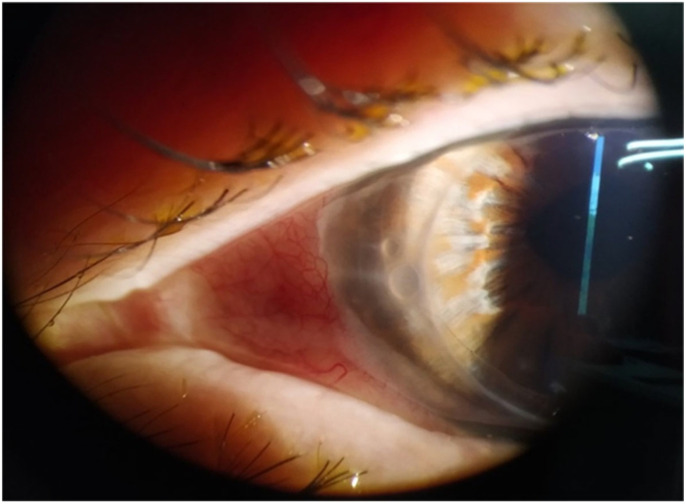
Three small bullae just outside the graft–host junction nasally.

Two days after his first dose of mRNA vaccination (Moderna Inc., Cambridge, MA, USA), he started having symptoms of photophobia and blurred vision in the left eye. His contralateral eye remained unaffected with a visual acuity of ETDRS 0.0 LogMAR (1.0 Snellen). In the affected eye, his best-corrected visual acuity (BCVA—pinhole) dropped to 0.65 LogMAR (0.22 Snellen) from 0.3 LogMAR (0.5 Snellen). Examination of the adnexa revealed no lesions. Comprehensive slit lamp examination revealed corneal endotheliitis with 3 small bullae in the vicinity (but outside) of the graft–host junction (Figure 1). Intraocular pressure (IOP) was normal, measuring 12 mmHg in both eyes, and dilated fundoscopy revealed no additional pathology.

### 2.2. Case 2

**Figure 2:**
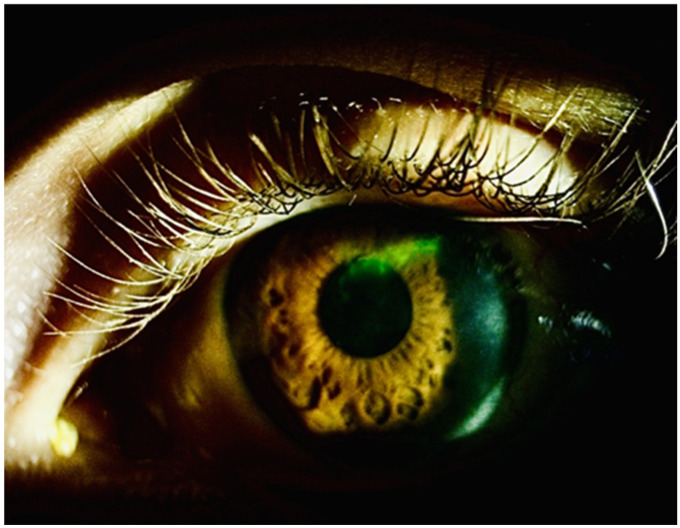
A dendritic ulcer is noted superiorly in the left eye.

A 24-year-old male student-athlete had recurrent herpetic keratitis in his left eye during the previous 18 months and was on prophylactic treatment of oral acyclovir 400 mg twice daily, keeping him free of recurrence for the previous 3 months. Three days after his first dose of vaccination (Pfizer Inc., Kalamazoo, MI, USA), he became symptomatic with photophobia and decreased visual acuity, thus seeking urgent review. His best-corrected visual acuity (BCVA) dropped to ETDRS 0.7 LogMAR (0.2 Snellen) from 0.1 LogMAR (0.8 Snellen) at his last follow-up 1 month prior to vaccination. Comprehensive slit lamp examination revealed recurrence of the epithelial disease with new dendrites (Figure 2) with an IOP of 14 mmHg in both eyes, and no evidence of retinitis, vasculitis or other pathology upon dilated fundoscopy. The patient being a student-scholarship athlete had no other medical history and was on no other medications.

**Figure 3:**
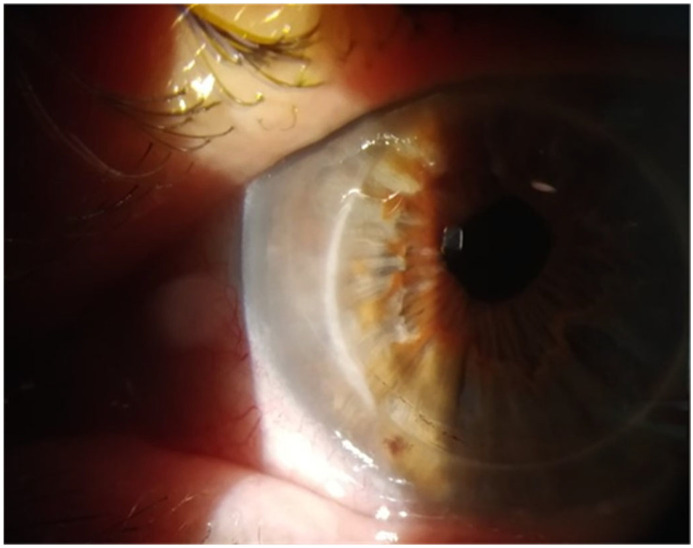
Complete resolution of the bullae.

**Figure 4:**
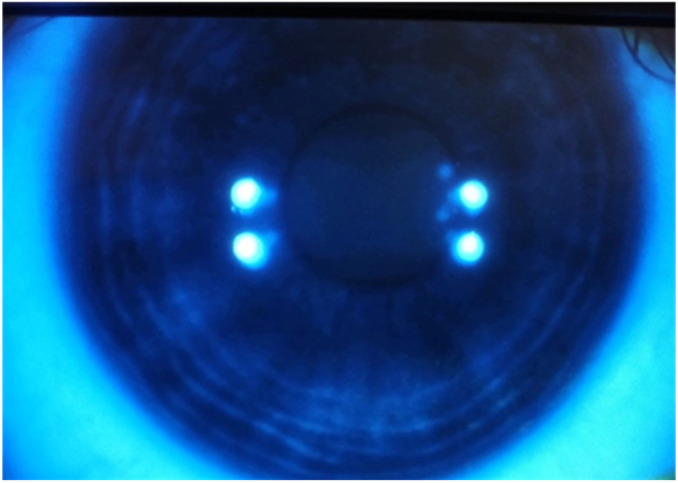
Completely healed dendritic ulcer.

The oral antiviral dose was increased in both cases from prophylactic to treatment levels of acyclovir 400 mg five times a day for one week. Within two days, we noted an improvement in symptoms and a significant reduction in the lesions, accompanied by symptomatic improvement experienced by the patients (Figure 3 and Figure 4). After one week of treatment, both patients returned to prophylactic antiviral dose. The grafted patient had the treatment with topical 0.1% dexamethasone (Thea, Clermont Ferrand, France) increased to four times a day, and after a week, continued with twice a day topical 0.1% dexamethasone (Thea, Clermont Ferrand, France). No topical antiviral was instituted for the second patient.

Two days prior to their second dose of the COVID-19 mRNA vaccine, both patients were advised to increase their antiviral regime to therapeutic levels for a week as a precaution. No relapse was observed, and they remain without recurrence to date.

## 3. Discussion

Herpetic keratitis recurrence has been noted a few days after the first dose of AstraZeneca and Pfizer-BioNTech COVID-19 mRNA vaccination (one and two cases, respectively). The first reported patient had a history of herpes simplex keratitis (quiescent for 40 years) with evidence of corneal scarring. He presented with typical corneal dendritic ulcer with worsening appearance despite topical treatment with ganciclovir gel. A diagnosis of herpetic stromal keratitis was made, requiring systemic aciclovir, topical prednisolone, moxifloxacin, atropine and oral doxycycline [5]. The other two patients were known to have herpetic keratitis and presented between 4 days and 4 weeks after receiving the vaccine [6]. One patient was diagnosed with necrotizing stromal keratitis, while the other with endotheliitis and epithelial keratitis. They both received systemic acyclovir and responded well to the provided treatment [6].

Further supporting these observations, a large-scale analysis of the NHIS database in Korea by Lee et al. (2025) investigated herpesviral keratitis following COVID-19 vaccination [7]. The study found a statistically significant but low incidence of reactivation, emphasizing the need for vigilance in high-risk patients, particularly those with a history of recurrent herpetic keratitis. Additionally, Kawahara (2025) reported two cases of herpes simplex keratitis reactivation, highlighting the potential role of vitamin D receptor agonists in modulating immune responses and possibly reducing recurrence rates in susceptible individuals without significant side-effects [8].

Possible immunologic mechanisms for reactivation of herpes simplex virus following vaccination include molecular mimicry whereby the immune system reacts to the vaccine but accidentally targets similar proteins in the body, triggering herpes reactivation; inflammation where the vaccine causes a strong immune response, releasing inflammatory substances (cytokines) that can activate dormant herpes viruses; and immune system stress when temporary changes in the immune system after vaccination might allow the herpes virus to reactivate [9]. Supporting the latter theory, a murine study demonstrated that intranasal infection with a non-neurotropic influenza virus led to decreased brain neurotrophin levels and microglial activation [10]. This decline in neurotrophic support may impair neuronal antiviral defenses, potentially enabling unchecked HSV replication and overwhelming immune surveillance in the CNS. Viral reactivation has been shown to occur after other vaccines, including Varicella zoster keratitis after Shingrix administration (GSK). Lu and Ta, 2022, described the reactivation of herpes zoster keratitis shortly after receiving the Shingrix vaccine [11]. The authors suggested that vaccine-induced inflammation or immune modulation might have triggered latent virus reactivation.

The initiation and duration of acyclovir prophylaxis for recurrent herpetic keratitis have remained a topic of controversy, particularly in the context of corneal transplants. HEDS II trial has guided our treatment of recurrent herpetic keratitis in the context of prophylaxis. This placebo-controlled trial enrolled 703 patients and demonstrated that daily oral acyclovir prophylaxis reduces the recurrence of both epithelial and stromal keratitis in patients with a history of ocular HSV, especially during the first year of use, by about 45–50% [12]. The treatment was generally well-tolerated, with no severe side effects directly attributed to acyclovir. However, some patients discontinued due to minor adverse effects like gastrointestinal discomfort. Long-term prophylactic use of acyclovir raises additional considerations. While it effectively reduces recurrence rates, prolonged use may contribute to antiviral resistance and requires careful evaluation of cost-effectiveness [12].

Long-term oral acyclovir therapy has been shown to significantly reduce recurrent infectious herpes simplex keratitis in patients with corneal grafts. Simon et al., 1996, highlighted the efficacy of the drug in suppressing viral reactivation, despite the potential for antiviral resistance to be raised by continuous prophylaxis [13].

The personal and public health benefits of COVID-19 vaccination are well documented [14]. Current literature shows a rare incidence of ocular adverse effects. The risk for patients to develop ocular adverse events in response to COVID-19 infection or vaccination remains remarkably low [15]. To the best of our knowledge, these are the first reported cases of reactivation of herpes simplex keratitis following vaccination for COVID-19 in defiance of antiherpetic prophylaxis. It is well-established that new risk factors of herpetic reactivation, such as resistance to acyclovir, may lead to new epithelial lesions or stromal/endothelial disease even during the period of prophylaxis [16]. While this observation warrants consideration, physicians should remain aware of the intricate interplay between HSV-1 and host immune responses, including the potential association between COVID-19 vaccination and HSV keratitis recurrence. Although evidence remains limited, our two cases suggest that patients on prophylactic antiviral therapy need close monitoring when experiencing even subtle symptoms of recurrence and may benefit from temporary dose escalation to therapeutic levels shortly after mRNA COVID-19 vaccination. Further research is needed to clarify this relationship and guide clinical management.
